# Unravelling Eribulin’s role in metastatic breast cancer: evaluating benefits for both triple negative and non-triple negative patients in real-world scenarios in resource-constrained settings

**DOI:** 10.3332/ecancer.2024.1804

**Published:** 2024-11-28

**Authors:** Akhil Kapoor, Anuj Gupta, Bipinesh Sansar, Pooja Gupta, Bal Krishna Mishra, Arpita Singh, Arvind Upadhyay, Amit Kumar, Mayank Tripathi, Zachariah Chowdhury, Shashikant Patne, Ipsita Dhal, Neha Singh, Shreya Shukla, Satyendra Narayan Singh, Lincoln Pujari, Prashanth Giridhar, Ankita Rungta Kapoor, Yash Jain, Manikandan Venkatachalam, Somnath Dey, Kunal Ranjan Vinayak

**Affiliations:** 1 Department of Medical Oncology, Mahamana Pandit Madan Mohan Malaviya Cancer Centre and Homi Bhabha Cancer Hospital, Tata Memorial Centre, Homi Bhabha National Institute, Varanasi 221005, India; 2 Department of Medical Oncology, Homi Bhabha Cancer Hospital and Research Centre, Tata Memorial Centre, Muzaffarpur 842004, India; 3 Department of Surgical Oncology, Mahamana Pandit Madan Mohan Malaviya Cancer Centre and Homi Bhabha Cancer Hospital, Tata Memorial Centre, Homi Bhabha National Institute, Varanasi 221005, India; 4 Department of Pathology, Mahamana Pandit Madan Mohan Malaviya Cancer Centre and Homi Bhabha Cancer Hospital, Tata Memorial Centre, Homi Bhabha National Institute, Varanasi 221005, India; 5 Department of Radiodiagnosis, Mahamana Pandit Madan Mohan Malaviya Cancer Centre and Homi Bhabha Cancer Hospital, Tata Memorial Centre, Homi Bhabha National Institute, Varanasi 221005, India; 6 Department of Radiation Oncology, Mahamana Pandit Madan Mohan Malaviya Cancer Centre and Homi Bhabha Cancer Hospital, Tata Memorial Centre, Homi Bhabha National Institute, Varanasi 221005, India; 7 Department of Nuclear Medicine, Mahamana Pandit Madan Mohan Malaviya Cancer Centre and Homi Bhabha Cancer Hospital, Tata Memorial Centre, Homi Bhabha National Institute, Varanasi 221005, India; 8 Department of Palliative Medicine, Mahamana Pandit Madan Mohan Malaviya Cancer Centre and Homi Bhabha Cancer Hospital, Tata Memorial Centre, Homi Bhabha National Institute, Varanasi 221005, India

**Keywords:** Eribulin, metastatic breast cancer, heavily pre-treated, response, toxicity

## Abstract

**Background:**

Metastatic breast cancer (MBC) patients have numerous options for treatment. However, it is essential to consider treatments with favorable toxicity profiles and convenient modes of administration. Eribulin has shown effectiveness in aggressive MBC, but there is a lack of sufficient real-world data specific to Indian patients.

**Patients and methods:**

We conducted a retrospective audit of patients with MBC who received intravenous Eribulin between 2019 and 2023 at a dosage of 1.4 mg/m^2^ on days 1 and 8 every 3 weeks. The median Progression-free survival (PFS) and overall survival (OS) were estimated using the Kaplan-Meier method.

**Results:**

During the specified time, 107 consecutive patients with MBC received Eribulin treatment. The median age was 52 years (range, 28–75 years) with 3 patients with male breast cancer. The median number of prior chemotherapy lines and involved sites were 3 (range, 2–5) and 3 (range, 1–6), respectively. Visceral involvement was present in 84 (78.5%) patients. A median of 3 cycles of Eribulin (range, 1–11) was administered. Eribulin resulted in partial responses in 49 (45.8%) patients, stable disease in 11 (10.3%) patients and progressive disease in 47 (43.9%) patients. The median PFS was 4.0 months (95% CI: 3.4–4.6), and the median OS was 10.0 months (95% CI: 8.3–11.7). For patients with triple-negative breast cancer (TNBC), the median OS was 8 months (95% CI: 5.6–10.4), whereas non-TNBC patients had a median OS of 11 months (95% CI: 9.1–12.8) (hazard ratio, 1.9, 95% CI: 1.2–3.1, *p* = 0.002). Eribulin was well-tolerated, with dose reduction was needed in 9 (8.4%) of the patients in the overall cohort.

**Conclusion:**

Eribulin is a viable and safe option for treating heavily pre-treated MBC in real-world settings. The study found comparable efficacy in both TNBC and non-TNBC patients.

## Introduction

Breast cancer (BC) is the most common cancer in India, with a 5-year prevalence of around 77.9 per 100,000 population as per GLOBOCAN 2022 data. It comprises of 26.6% of all cancer incidences and 13.7% of all cancer-related mortality in India [1, 2]. The incidence of metastatic breast cancer (MBC) has been reported to be between 5% and 25% in different Indian cancer registries [3]. Of these, around 5%–10% of patients present with upfront metastasis, while 20%–30% develop metastasis during their regular follow up [3]. Triple negative MBC has a dismal prognosis globally though more so in India with 5 and 10-year survival to the tune of 22% and 5%, respectively [3–5]. Access to newer drugs like Sacituzumab govitecan or precision medicine approaches are not possible due to the financial toxicity attached to their use [5]. Heavily pre-treated MBC is difficult to manage owing to the compromised marrow reserve and residual toxicities and infrastructure limitations in low and middle-income countries (LMICs) further complicate it. Traditionally, anthracycline and taxanes are the backbone of treatment in BC while in MBC, various drugs like gemcitabine [6], liposomal doxorubicin [7] and capecitabine, [7, 8] have been used with responses around 10%–30%.

Eribulin is a non-taxane microtubule inhibitor which is a Halichondrin B analog and it acts by inhibiting the formation of mitotic spindles causing mitotic arrest in the G2M phase [9]. Eribulin is known have its effect on vascular remodelling and increasing subsequent administered drug delivery. Besides, it is known to activate mesenchymal to epithelial phenotype switch by inhibiting transforming growth factor TGF beta, and facilitating favourable immune microenvironment [10]. All these mechanisms make eribulin a promising drug in MBC. The phase 3 EMBRACE study has shown a significant overall survival (OS) benefit in pre-treated MBC compared to physician’s choice and is approved for use in MBC post 2 lines of treatment [12, 13]. Nevertheless, there is a paucity of real-world data from the LMICs including India. Therefore, we planned to do this study at our centre to assess the efficacy and safety of Eribulin in real-world settings.

## Patients and methods

This is a single-centre retrospective audit of MBC patients who received Eribulin between January 2019 and July 2023. Patients were classified as per the standard hazard ratio (HR+), Her2+ or triple negative breast cancer (TNBC). Patients who expressed both HR and Her2, were treated as per Her2 positive disease and hormonal treatment was added in the maintenance treatment along with Her2 treatment, as per the standard institutional protocol. The eligible patients were men or, women ≥18 years of age with histologically confirmed BC, an Eastern Cooperative Oncology Group performance status (ECOG PS) of 0–2, and adequate bone marrow, liver and renal function. The patients must have received at least two prior lines either in the adjuvant or in the metastatic setting. The patients should have progressed on anthracyclines and taxanes. TNBC patients must have progressed on platinum-based chemotherapy as well. The patients received intravenous eribulin at a dose of 1.4 mg/m^2^ over 3–5 minutes on day 1 and 8 of 3 weekly schedule with prophylactic growth factor support after day 1 and 8 doses for 3 days each. Patients who were Her2 positive, received trastuzumab in the standard dosage along with Eribulin. Eribulin was continued until disease progression or unacceptable toxicity. Response assessment was performed after every 3–4 cycles, or earlier as per the physician discretion and clinical situation. The toxicities were recorded as per CTCAE 5.0 criteria.

Duration of eribulin therapy was measured from the date of start of eribulin until discontinuation for any reason. Progression free survival (PFS) and OS were measured from the date of start of eribulin to the date of progression and death, respectively. The patients were censored at the date of last follow up. The data were collected from the electronic medical records of the hospital and was entered in an excel sheet for further analysis.

Since the patients were treated with standard institutional protocol and there was no direct contact between the researcher and the patients for the purpose of this study, the need for informed consent was waived. The institutional review board approved the study vide letter number OIEC/11000817/2024/0000.

### Statistical analysis

The data were analysed using the Statistical Package for the Social Sciences (SPSS version 20, Armonk, NY; IBM Corp). Continuous variables were described using the median, whereas the non-continuous variables were described using proportions. Kaplan Meier method was used for calculating PFS and OS. The Schoenfeld plots were made for each variable to check the assumption of proportionality in R software (R Core Team, Vienna, Austria). Cox regression analysis was conducted to calculate HR by log-rank test. The same method was also used to calculate the HR of various prognostic factors for their effect on OS.

## Results

### Baseline demographics

One hundred fifteen patients of MBC were planned for treatment with eribulin, however, there was no data of the actual delivery of eribulin to 8 patients, thus, these were excluded from the analysis. 107 patients received eribulin over the planned study period and all these patients were included for analysis. The baseline demographics of the patients are summarised in Table 1. The median age was 52 (range, 28–75) years. The majority (*n* = 102, 95.3%) of the patients had ECOG-PS <2. Estrogen receptor (ER) TNBC were equally distributed (48.6% and 45.8%, respectively). Most of the patients (*n* = 80, 74.8%) had metastasis to more than 2 sites. Germline BRCA testing was performed in 21 TNBC patients, out of which 5 (23.8%) came as BRCA1/2 positive. Three of these patients received Olaparib therapy as well as a prior line of therapy before eribulin. All TNBC patients had received platinum-based chemotherapy earlier (carboplatin in combination with paclitaxel or, gemcitabine). CDK4/6 inhibitors were given to 31 (59.6%) of the ER+ patients in prior lines. At the time of the start of eribulin, the most common site of metastasis was bone (61.7%). The median number of cycles of eribulin received was 3 (range 1–11).

### Outcome analysis

**Figure 1.**
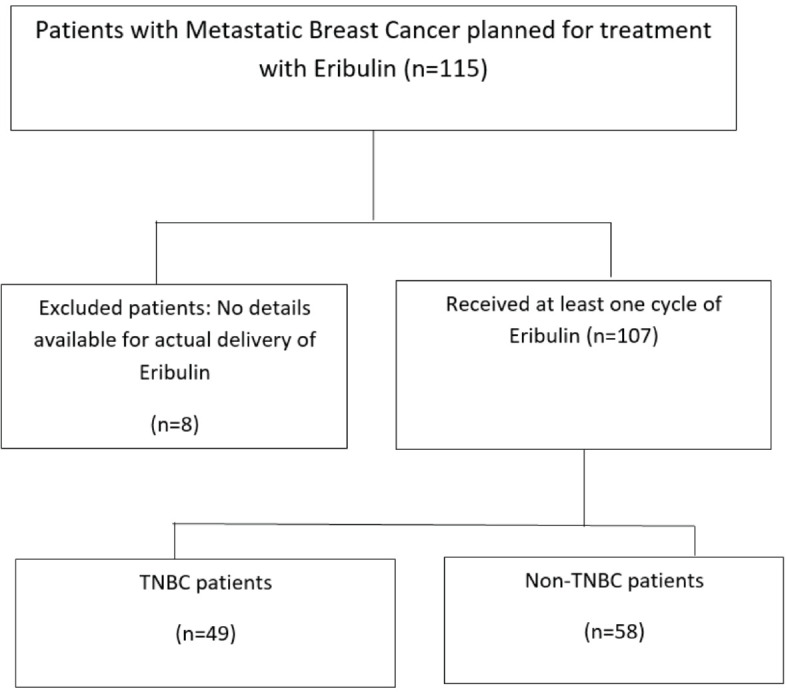
Consort diagram of the study.

**Figure 2.**
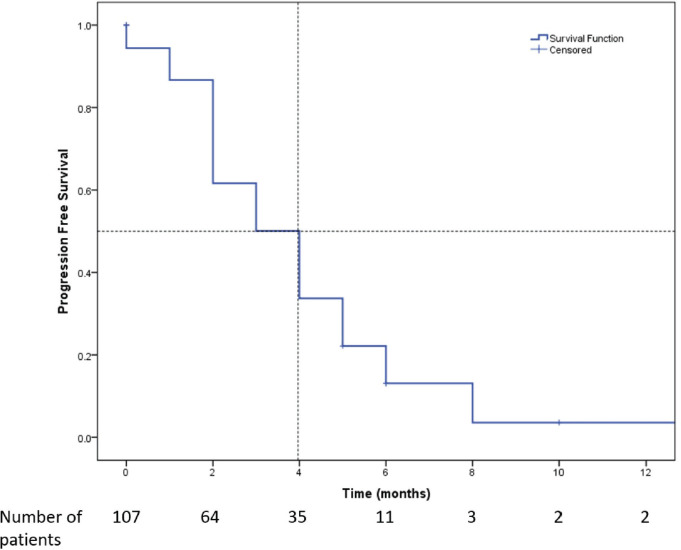
Kaplan-Meier survival curve showing the PFS of the patients who received Eribulin (*n* = 107). Median PFS was 4 months (95% CI: 3.4–4.6).

**Figure 3.**
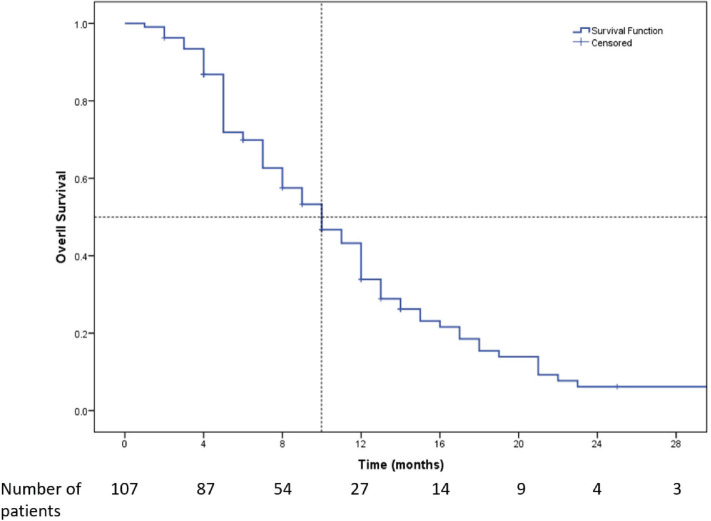
Kaplan-Meier survival curve showing the OS of the patients who received Eribulin (*n* = 107). Median OS was 10 months (95% CI: 8.3–11.7).

**Figure 4.**
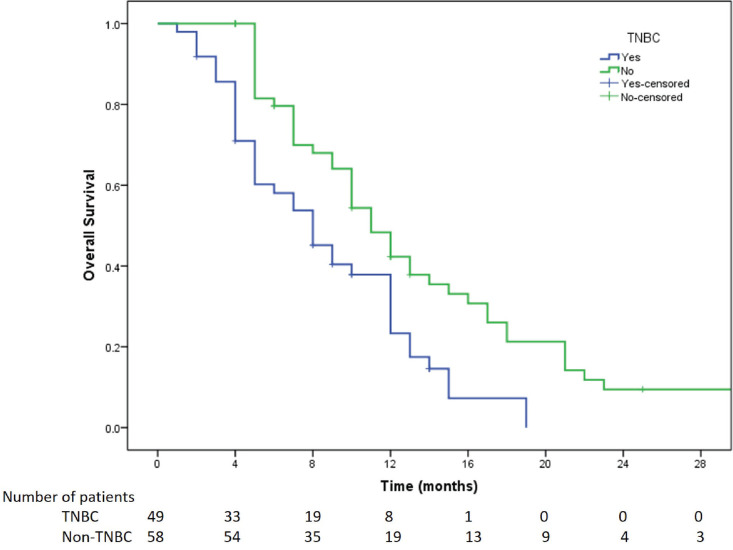
Kaplan Meier survival curve showing the OS of TNBC versus non-TNBC patients included in the study.

All the 107 patients were evaluated for response. Figure 1 shows the consort diagram of the study. The best response as per RECIST 1.1 was evaluated. Of all of the patients receiving eribulin, partial response (PR) was seen in 49 (45.8%) patients, while stable disease (SD) was observed in 11 (10.3%) patients. In 47 (43.9%) patients, there was disease progression (PD) as the best response to eribulin. The outcome data are summarised in Table 2. The median PFS on eribulin was 4 months (95% CI: 3.4–4.6, Figure 2), while the median OS was 10 months (95% CI: 8.3–11.7, Figure 3). Post eribulin treatment, 58 (54.2%) patients received other chemotherapy, while, the remaining 49 (45.8%) patients were planned for supportive care alone. Since the Schoenfeld plots nearly represented a horizontal line, it was inferred that the data do not violate the proportional hazard assumption. Thus, cox regression analysis of the prognostic factors was carried out. The analysis revealed patients having TNBC and having more than three metastatic sites had poorer outcomes, both the factors retaining significance in both univariate and multivariate analysis (Table 3). Patients with TNBC had median OS of 8 months (95% CI 5.6–10.4) as compared to 11 months (95% CI 9.1–12.8) in non-TNBC patients, HR 1.9 (95% CI: 1.2–3.1, *p* = 0.002, Figure 4).

### Toxicity analysis

Eribulin was well tolerated with only 5 (4.6%) patients discontinuing therapy due to grade 4 febrile neutropenia in 4 and drug-induced lung injury in 1 patient. Table 4 provides the details of the toxicities on Eribulin treatment. Significant grade 3/4 hematological toxicities were seen in 21.5% (23/107) patients. Besides haematological toxicity, grade 3/4 neuropathy and mucositis were seen in 6 (5.6%) and 4 (3.7%) patients, respectively while grade ¾ transaminitis in 4 (3.7%) of the patients. Dose reduction was needed in 9 (8.4%) of the patients in the overall cohort.

## Discussion

There is an unmet need to improve the survival of patients in MBC specially in heavily pre-treated patients. Multiple options have been tried in third-line and fourth-line treatment post anthracyclines and taxanes. There is evidence that upto 40% of patients may have a clinically meaningful benefit in third-line setting in MBC. However, due to a lack of robust evidence, there is no consensus to which drug is to be preferred in the third line and beyond. Multiple trials have shown that monotherapy with capecitabine has a response rate of 19%, ixabepilone has 11.5% and nab-paclitaxel has around 30%. Another commonly used regimen in real-world settings is gemcitabine carboplatin, which has disease control rates of around 45% [11]. The EMBRACE and study 301 along with limited data from Asia has shown good efficacy of eribulin monotherapy. The phase 3 EMBRACE study has shown a significant OS benefit in pre-treated MBC compared to physician’s choice (13.2 versus 10.5 months, HR 0:81; *p* = 0.014) and is approved for the use in MBC post 2 lines of treatment [12, 13].

Our study provides the largest real-world Indian data of 107 patients with a relatively younger cohort (median age of 52 years) who were heavily pre-treated with median lines of therapy 3 (2–5). Most of them had a heavy disease burden with more than two organ involvement in 74.8% of patients. The cohort was predominantly comprised of HR positive (48.6%) and TNBCs (45.8%) patients, while only 5.6% were Her2 positive. The PFS and OS in our study were comparable to other studies and slightly inferior statistics are attributed to the fact that patients had very high disease burden [11, 12]. Another notable finding in this study being the better survival in non-TNBC patients as against the usual report of better efficacy of eribulin in TNBC patients [12]. This could stem from a small sample size leading to artifact and TNBC patients having multiple sites of visceral metastasis leading to poorer outcomes (Table 5). However, this real world data gives confidence in utilising eribulin in non-TNBC patients as well. Twelves *et al* [14] reported that eribulin improves OS in various patient subgroups with MBC who had previously received an anthracycline and a taxane. Our study found that patients having TNBC and having more than three metastatic sites had poorer outcomes, which highlights the poor prognosis associated with these subgroups.

The limitations of this study were the retrospective design, and single institute nature with inherent biases; however, this is the real-world data of therapy which is now increasingly being utilised in MBC, primarily due to a reduction in the cost of this therapy.

## Conclusion

Eribulin is a viable and safe option for treating heavily pre-treated MBC in real-world settings. The study found comparable efficacy in both TNBC and non-TNBC patients.

## Conflicts of interest

Nil.

## Funding

No funds were received for the purpose of this study.

## Figures and Tables

**Table 1.** Baseline characteristics of the patients.

Details	*N* (Percentage)
Age (median, range)	52 years (28–75 years)
Gender Female Male	104 (97.2%) 3 (2.8%)
ECOG-PS 1 2	102 (95.3%) 5 (4.7%)
Comorbidities None Present	80 (74.8%) 27 (25.2%)
Receptor status ER/PR Positive Her2 positive TNBC	52 (48.6%) 6 (5.6%) 49 (45.8%)
Sites of disease Liver Lung Pleural effusion Brain Bones Nodes Others	46 (43.0%) 55 (51.4%) 23 (21.5%) 23 (21.5%) 66 (61.7%) 51 (47.6%) 10 *(9.3%)
Number of metastatic sites ≤ 3 >3	Median 3 (range 1–6) 35 (32.7%) 72 (67.3%)
Number of lines of prior therapy	Median 3 (range 2–5)
Lines of therapy before Eribulin 2–3 >3	89 (83.2%) 18 (16.8%)

*Others include- (Adrenal-2, Ascites-3, Bone Marrow-3, omentum-2)

**Table 2.** Response and outcomes of the use of Eribulin.

Best response post Eribulin (based on imaging)	PR-49 (45.8%) SD-11 (10.3%) PD-47 (43.9%)
Number of cycles of Eribulin	3 (range-1–11)
Treatment post Eribulin	Best supportive care alone- 49 (45.8%) Chemotherapy- 58 (54.2%)

**Table 3.** Cox regression model of the known prognostic factors for OS.

Feature	HR	95% confidence interval	*p* value
TNBC versus non-TNBC	2.1	1.3–3.3	0.002
Elderly versus non-elderly	0.9	0.5–1.6	0.816
Number of metastatic sites (upto 3 versus more than 3)	1.3	1.1–1.6	0.004
Liver metastases versus no liver metastases	0.8	0.5–1.3	0.372
Brain metastases versus no brain metastases	0.7	0.4–1.4	0.331
Visceral versus no visceral disease	1.2	0.6–2.3	0.557

Note: Elderly was defined as age more than 60 years

**Table 4.** Toxicities with Eribulin treatment.

Toxicity	All grades	Grade 3–4
Anemia	24 (22.4%)	9 (8.4%)
Thrombocytopenia	14 (13.0%)	7 (6.5%)
Neutropenia	25 (23.4%)	7 (6.5%)
Mucositis	62 (57.9%)	4 (3.7%)
CINV	11 (10.3%)	0
Neuropathy	69 (64.5%)	6 (5.6%)
Transaminitis	12 (11.2%)	4 (3.7%)
Fatigue	14 (13.1%)	0
Others*	3 (2.8%)	1 (0.9%)
Dose modification	9 (8.4%)	

*Others (1-diarrhea, 1-drug induced lung injury, 1-rash, 1-myopathy), CINV- chemotherapy induced nausea and vomiting

**Table 5.** Comparison with other studies with Eribulin monotherapy.

Study	*n*	Median PFS (months)	Median OS (months)
EMBRACE study	762	3.7	13.1
Study 301	1,102	4.1	15.9
Sirven M.B study	95	4.1	11.1
Aogi *et al* study	80	3.9	11.1
Present Study	107	4.0	10.0
